# Royal British Nurses' Association

**Published:** 1920-06-26

**Authors:** 


					Royal British Nurses' Association
The annual meeting of the members of this Associa^0
was held in the rooms of the Medical Society of Lob"1 ,
on June 21, under the chairmanship of Mrs. Camp''8'
Thomson.
The Medical Hon. Secretary (Mr. Herbert J. Patef^
F.R.C.S.) read the annual report. This opened wi^,.
congratulatory reference to that landmark in the aB^,
of nursing, the registration of trained nurses. ^
March a number of nurses who were interested
health work decided to form an Association of Tr^1 A
Nurses in Public Health work, and it had already
useful work in pressing for the employment of fully-tr?1 ^
nurses in public health activities; it had been affile,
to this Association. The Society of Chartered Nu'
was reconstituted during the year, and its nurses
had representation on its Committee of Management,
formation of a club could not be accomplished yet,
to pressure of work, but it was hoped to do so ^
a reasonable time. Mr. Paterson concluded with a V
of warm appreciation of the labours of Miss Macdo11
as Secretary. He moved the adoption of the rep"
which was seconded by Miss Holmes.
Miss Breay thought the Association could congrat?
itself on the fact that the ignoble policy of the Co
of Nursing, which was referred to in the report;
not succeed. Had it not been for the Minister of S ^
subsequently bringing in a Government Bill the sick ^
the suffering, as well as the nurses, might have ^
for another quarter of a century for their Bill.
Dr. Kenneth Stewart (Hon. Treasurer) read ^
financial report, which showed a balance of ?50 ^s' ;
on the wrong side, largely due to the enhanced c? J
printing and pressure of work at the office. The?6
also a reduction in donations by ?379.
Elections to the Council. jj<
The Secretary read the result of the election to fi'y
vacancies on the Council as follows : Colonel Ope^5 j)l
Inspector-General Woods, Mr. Murray, Dr. Lord, y
Glover, Dr. Belfrage, Dr. McEwen, Dr. Anderson*^
Domville, Dr. Outterson Wood, Miss Bushby.
Donaldson, Miss Dowbiggin, Miss Hurlston, Miss v.a
Miss Murby, Miss Vergette, Miss Montgomery-^, uf
Miss Reeves, Miss Ford, Miss Rossiter, Miss -^a W
Miss Gunn, Miss Bennett, Miss Stoddart, Mrs. SheIw;
Miss Ayres, Miss Stewart Murray, Miss Wise, Miss

				

